# Improving anticoagulation in sub‐Saharan Africa: What are the challenges and how can we overcome them?

**DOI:** 10.1111/bcp.14768

**Published:** 2021-03-10

**Authors:** Johannes P. Mouton, Marc Blockman, Christine Sekaggya‐Wiltshire, Jerome Semakula, Catriona Waitt, Munir Pirmohamed, Karen Cohen

**Affiliations:** ^1^ Division of Clinical Pharmacology, Department of Medicine University of Cape Town Cape Town South Africa; ^2^ Infectious Diseases Institute, College of Health Sciences Makerere University Kampala Uganda; ^3^ Department of Molecular and Clinical Pharmacology, Institute of Translational Medicine University of Liverpool Liverpool UK; ^4^ Wolfson Centre for Personalised Medicine, Institute of Translational Medicine University of Liverpool Liverpool UK

**Keywords:** anticoagulants, cardiovascular disease, drug utilization, medication safety

## Abstract

Patients in sub‐Saharan Africa generally have poor anticoagulation control. We review the potential reasons for this poor control, as well as the potential solutions. Challenges include the affordability and centralisation of anticoagulation care, problems with access to medicines and international normalised ratio monitoring, the lack of locally validated standardized dosing protocols, and low levels of anticoagulation knowledge among healthcare workers and patients. Increasing numbers of patients will need anticoagulation in the future because of the increasing burden of noncommunicable disease in the region. We propose that locally developed “warfarin care bundles” which address multiple anticoagulation challenges in combination may be the most appropriate solution in this setting currently.

## 1 INTRODUCTION

Anticoagulation is used to treat and prevent venous thrombosis and to prevent intracardiac thrombosis due to some structural heart diseases or dysrhythmias. Depending on the indication, treatment may be lifelong. Over‐anticoagulation may result in bleeding and under‐coagulation in thrombotic complications, including stroke. With the advent of direct oral anticoagulants (DOACs), there is increased choice of drugs available. However, vitamin K antagonists such as **warfarin** remain the most widely used oral anticoagulants and form the focus of this article.

When using vitamin K antagonists, the degree of anticoagulation is measured by the international normalised ratio (INR), and a patient's longitudinal anticoagulation control can be described by the proportion of INRs which fall in the therapeutic range (PTR), or by the proportion of time spent in the therapeutic range (TTR), interpolating INR results for the time between actual INR measurements. Patients in sub‐Saharan Africa (SSA) have poor anticoagulation control as measured by their PTR/TTR. For example, under trial conditions South African participants in three large trials had mean TTRs of 55%,^1^ 46%^2^ and 58%.^3^ In an atrial fibrillation (AF) registry mean TTR in nine African countries was 33% (*vs* 62% in 19 Western European countries)^4^ and in a rheumatic heart disease registry predominantly conducted in African countries PTR was 28%.^5^ A number of smaller observational studies from SSA are presented in Table 1; in all but one of these PTR or TTR ranged between 28% and 52%.

**TABLE 1:** Observational studies from sub‐Saharan Africa which measured time in therapeutic range (TTR) or proportion of INR results in therapeutic range (PTR) among patients on anticoagulation

Study, publication year	Setting	Sample characteristics	Sample size	Mean or median TTR	Mean or median PTR
Makubi,^6^ 2008	Dar Es Salaam, Tanzania National referral hospital	Patients with mechanical heart valves attending the ACC	189		36%
Manji,^7^ 2011	Eldoret, Kenya Teaching hospital	Adults attending ACC	178	65%	
Menanga,^8^ 2015	Yaoundé, Cameroon A general and a central hospital	Adult inpatients and outpatients with AF on VKA	27	48%	
Daba,^9^ 2016	Addis Ababa, Ethiopia Teaching hospital	Adults with VTE on anticoagulation	91		34%
Mariita,^10^ 2016	Nairobi, Kenya Teaching hospital	Adults attending cardiac, cardiothoracic or haemato‐oncology clinic for anticoagulation monitoring	147		44%
Sadhabiriss,^11^ 2016	Durban, South Africa District‐level hospital	Patients with nonvalvular AF or prosthetic heart valves attending outpatient department on warfarin for ≥12 mo	177	30%	
Sonuga,^12^ 2016	Cape Town, South Africa District hospital	Adults attending ACC and on warfarin for ≥30 d	136		49%
Ahmed,^13^ 2017	Khartoum, Sudan Specialist hospital	Adults attending ACC and on warfarin for ≥1 yr	135		52%
Fenta,^14^ 2017	Addis Ababa, Ethiopia Teaching hospital	Adults attending cardiology or haematology outpatient clinics on warfarin for ≥3 mo	360		29%
Nyamu,^15^ 2017	Nairobi, Kenya Teaching hospital	Adults attending cardiac, cardiothoracic or haemato‐oncology clinics on warfarin	102		28%
Coulibaly,^16^ 2018	Abidjan, Côte d'Ivoire Specialist hospital	Adults with nonvalvular AF on VKA for ≥6 mo and with ≥6 INR measurements	100		44%
Ebrahim,^17^ 2018	Cape Town, South Africa A specialist hospital and a community health centre	Patients attending two ACCs and on warfarin for ≥27 mo with regular INR monitoring	363	47%	
Mwita,^18^ 2018	Gaborone, Botswana Tertiary hospital	Patients attending medical outpatient clinics on warfarin for ≥1 mo with ≥4 INR measurements	410	31%	
Jonkman,^19^ 2019	Windhoek, Namibia Central hospital	Patients attending ACC with ≥3 INR values	215	29%	25%
Karuri,^20^ 2019	Nairobi, Kenya Teaching hospital	Patients attending cardiac, cardiothoracic or haemato‐oncology clinics on warfarin for ≥1 mo with ≥2 INR measurements	406	31%	
Botsile,^21^ 2020	Gaborone, Botswana Tertiary hospital	Adults with mechanical heart valves attending ACC on warfarin for ≥1 mo with ≥3 INR measurements	142	30%	
Semakula,^22^ 2020	Kampala, Uganda and Cape Town, South Africa A primary level health centre and four secondary and tertiary level hospitals	Patients attending five ACCs	229	41%	

ACC, anticoagulation clinic; AF, atrial fibrillation; INR, international normalised ratio; PTR, proportion of INR results in therapeutic range; TTR, time in therapeutic range; VKA, vitamin K antagonist; VTE, venous thromboembolism.

In this review, we explore potential reasons for this poor anticoagulation control in SSA and explore strategies to overcome these challenges.

## 2 METHODS

For this narrative (nonsystematic) review, we searched Pubmed and Africa‐Wide Information through EBSCOhost, using combinations of index terms (eg, “anticoagulants”, “Africa South of the Sahara”) and free text (eg, “anticoagulation”, “warfarin”, individual country names). We retained citations clustering around four themes: (1) burden of anticoagulation indications; (2) challenges accessing anticoagulation; (3) challenges with selecting and adjusting anticoagulant dose; and (4) patient‐related challenges including adherence, knowledge, attitudes and beliefs, and genetic factors. We also searched Google and dissertation databases (ProQuest Dissertations & Theses A&I, Open Access Theses and Dissertations (**oatd.org**)) for grey literature from SSA on these issues. Further reports were found by handsearching reference lists of included studies. Searches were conducted between March and May 2020.

## 3 CHANGING LIFE EXPECTANCY AND DISEASE BURDEN

**FIGURE 1:**
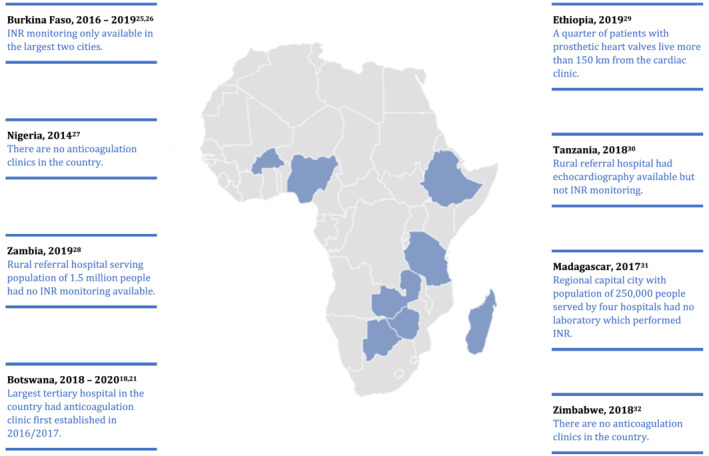
Example observational studies from sub‐Saharan Africa which describe resource limitations

SSA is undergoing an epidemiological transition: with increasing life expectancy the increasing burden of noncommunicable disease is colliding with the pre‐existing burden of infectious diseases. In the context of anticoagulation, this transition is evident in the increasing prevalence of (nonvalvular) AF, adding to the large number of people requiring anticoagulation for valvular heart disease, which in SSA is still mostly caused by rheumatic heart disease.^23^ This increase in indications for anticoagulation is occurring amidst an uneven distribution of limited resources. For example, there were only 57 centres in SSA capable of performing regular open‐heart operations in 2011/2012 (one per 15 million population); 35 of these were in South Africa.^24^ Figure 1 shows some examples of SSA anticoagulation studies mentioning resource limitations.

A recent systematic review suggested that AF prevalence in SSA may be higher than previously thought,^33^ up to 4.3% in one Ethiopian community‐based survey.^34^ Patients with AF in SSA have high prevalence of concomitant stroke risk factors^33^, ^35^ and should therefore benefit from anticoagulation. However, despite clear indications for anticoagulation, it is not always implemented in SSA.^26^, ^36^, ^37^, ^38^, ^39^, ^40^, ^41^ Indeed, in the multiregional RELY‐AF registry,^4^ patients in Africa had the second‐lowest use of oral anticoagulation where indicated (19%, second only to China).

Rheumatic heart disease prevalence among school children in SSA is 1.5‐3.0%.^42^, ^43^, ^44^, ^45^, ^46^, ^47^ In rheumatic heart disease registries, only two‐thirds of patients with an indication for oral anticoagulation received it.^5^, ^37^, ^48^

Venous thromboembolism (VTE) epidemiology in SSA has not been well described,^49^ but HIV infection is a well‐established risk factor, associated with a 1.5‐fold increased hazard (95% confidence interval 1.1 to 2.0).^50^ As the HIV pandemic epicentre, HIV‐associated VTE is commonly seen in SSA. In all the studies in Table 2, HIV prevalence was higher among patients presenting with VTE than the background prevalence. Interestingly, in a Ugandan study 9% of patients on antiretroviral therapy attending routine outpatient follow‐up were found to have incidental deep venous thrombosis.^51^

**TABLE 2:** Studies from sub‐Saharan Africa reporting the prevalence of HIV and tuberculosis among patients presenting to hospitals for venous thromboembolism

Study	Setting	Population HIV prevalence^a^	Proportion HIV infected	Proportion TB infected
Mampuya^52^	Regional/tertiary hospital, Kimberley, Northern Cape, South Africa, 2010‐2014	11.0%	443/852 (52%)	106/852 (12%)
Louw^53^	Tertiary hospital, Johannesburg, Gauteng, South Africa, date NR	16.9%	11/24 (46%)	NR
Goldstein^54^	Tertiary hospital emergency centre, Johannesburg, Gauteng, South Africa, 2012‐2013	17.8%	35/70 (50%)	21/70 (30%)
Awolesi^55^	Urban district hospital, KwaZulu‐Natal, South Africa, 2013	27.0%	42/81 (52%)	29/81 (36%)
Olubanwo^56^	Tertiary hospital, Mthatha, Eastern Cape, South Africa, 2010	18.0%	81/102 (79%)	42/102 (41%)
Alshehri^57^	District hospital, Cape Town, Western Cape, South Africa, 2008‐2011	8.9%	393/610 (64%)	339/610 (56%)
Kamdem^58^	Tertiary urban hospital, Douala, Cameroon, 2008‐2016	3.6%	11/78 (14%)	3/78 (3.8%)
Nkoke^59^	Semi‐urban regional hospital, Buea, Cameroon, 2016‐2017	3.6%	5/22 (23%)	4/22 (18%)
Abah^60^	Semi‐urban military hospital, Bamenda, Cameroon, 2010‐2013	3.6%	17/79 (22%)	NR
Ogeng'o^61^	National referral hospital, Nairobi, Kenya, date NR	4.7%	14/128 (11%)	16/128 (13%)

^a^ HIV prevalence among adults 15‐49 years old. For South Africa, these are provincial estimates from the Thembisa model^62^ for the final year of study data collection (or publication, if not reported). For other countries, these are 2018 national estimates by UNAIDS.^63^

## 4 CHALLENGES ACCESSING ANTICOAGULATION

Vitamin K antagonists appear on most SSA countries' essential medicine lists.^64^ Warfarin is the most commonly used, followed by acenocoumarol.^64^, ^65^ Warfarin is cheap: a 5 mg tablet cost 8 US cents in rural Zambia^59^ or 14 US cents in Uganda.^66^ However, being essential and cheap does not necessarily make medicines available or accessible.^67^ Essential medicines' availability ranged from 25% in public facilities to 49% in private facilities in Cameroon,^68^ and from 49% in public facilities to 71% in retail pharmacies in Malawi.^69^ Anecdotally, frequent stock‐outs of warfarin have been mentioned in some SSA studies.^22^, ^29^, ^70^ To our knowledge, the only study to systematically evaluate warfarin availability in SSA was conducted in Uganda in 2017^66^ and showed that warfarin was available on the survey day at 75/100 private pharmacies, 15/23 (65%) private hospitals and only 4/22 (18%) public hospitals, all randomly sampled.^66^

DOACs were only available in 14/33 African countries surveyed in 2018,^71^ and are often beyond the means of patients and public health services. In South Africa, which has statutory private sector medicine ceiling prices, 1 month's supply of dabigatran, apixaban or rivaroxaban costs the equivalent of approximately 60 hours' minimum wage.^72^ To our knowledge, no DOAC has yet been shown to be cost‐effective in any SSA country's public health sector.^73^

A weak medicines regulatory environment in much of SSA means that substandard and falsified medicines may be found on the market.^74^ We are not aware of any SSA data on the quality of warfarin on the market, but in a medicines quality assessment across 10 West and Central African countries, there was fortunately no evidence of poor quality acenocoumarol.^75^

Dedicated anticoagulation clinics using standardized approaches may achieve better anticoagulation control than routine models of care, where anticoagulation patients are seen as part of the general patient mix.^76^, ^77^, ^78^, ^79^, ^80^, ^81^ Such anticoagulation clinics are, however, not common in SSA; anticoagulation is often managed in outpatient cardiology, cardio‐thoracic surgery and haemato‐oncology clinics^20^, ^32^, ^82^ or by individual healthcare workers who may not use standardized approaches.^27^, ^83^ Prescribers of anticoagulation are often junior with limited practice experience.^83^

A few studies investigated SSA healthcare workers' knowledge, attitudes and beliefs about anticoagulation. In one such study 164 doctors and pharmacists at an Ethiopian tertiary hospital completed a self‐administered questionnaire.^84^ Participants' mean score on the warfarin knowledge section was 10/15 correct answers, yet only 7% identified their own lack of knowledge on warfarin as a barrier to effective patient counselling.^84^ Specific knowledge gaps identified included drug‐drug interactions and the target INR range appropriate to specific indications.^84^ In a second study at the same hospital, investigators directly observed the counselling pharmacists provided during warfarin dispensing and found that only 10% of patients were told what to do when they missed a dose, while interactions and side effects were discussed in only 9% and 3% of encounters, respectively.^85^ In addition, just 24% of patients were given an opportunity to ask questions at the dispensing encounter and only 40% of warfarin containers were labelled.^85^ At a South African academic hospital, in 86% of admissions for over‐anticoagulation with warfarin the cause of toxicity was not identified by attending clinicians.^86^ Drug‐drug interactions with warfarin, which went unrecognised by the attending clinicians, were retrospectively identified by study investigators in 77% of these patients.^86^

INR testing in centralised laboratories is the norm across SSA. As a result, people living in rural settings often have no access to INR monitoring. However, centralisation can even bypass people in urban settings, as illustrated in some of the examples in Figure 1. Centralised laboratory INR testing also means that results are delivered with a long turn‐around time, often a day or two,^22^, ^27^ and that patients may be required to attend separate blood sampling and INR monitoring visits,^22^, ^87^ driving up the expenses they incur.

The cost of INR testing has been identified as a barrier to anticoagulation therapy in Zambian^88^ and Ethiopian reports.^29^ We are aware of only one study systematically investigating the availability and cost of coagulation profile testing in SSA, conducted in Uganda in 2017.^66^ At randomly sampled facilities, coagulation profile testing was available at 13/22 (59%) private hospitals and 3/22 (14%) public hospitals,^66^ at a median price of US$8.30.

At least two different point‐of‐care INR monitors have been validated in South African patients,^89^, ^90^, ^91^ and there have been reports of using point‐of‐care INR monitors in anticoagulation clinics in Namibia,^19^ Kenya^7^, ^70^, ^92^, ^93^ and Nigeria.^87^ Considering only the cost per test, point‐of‐care testing is more expensive than laboratory monitoring; however, it may be cost‐effective in some settings, for example rural and remote settings.^94^ In SSA, most sites that reported using point‐of‐care testing received the monitors and test strips through donations, and offered point‐of‐care tests at a subsidised cost to patients.^7^, ^87^, ^93^

## 5 CHALLENGES WITH DOSE SELECTION AND DOSE ADJUSTMENTS

Warfarin dose selection and adjustment can be divided into an initiation phase, until a stable dose and INR is achieved, and a maintenance phase, during which further dose adjustments may be required for clinical and dietary reasons. Examples of dosing guidelines made by ministries/departments of health in SSA countries are given in Table 3. Except for the South African guideline, these are vague, with a large range of warfarin initiation doses and little detail about how often or by how much doses should be adjusted. Aside from these national guidelines, institution‐specific anticoagulation protocols, guidelines or algorithms may be in use: in one Nigerian survey 11% of clinicians reported using such guidelines in their institutions.^95^ At the time of our search, we did not find any warfarin dose initiation or dose adjustment algorithms in use in SSA that have been validated for the local population; our group subsequently published a validated dose initiation algorithm developed in South African and Ugandan patients.^96^

**TABLE 3:** Examples of warfarin dose initiation and dose adjustment guidelines for venous thromboembolism by SSA countries' departments or ministries of health

Guideline	Warfarin dose initiation guideline	Warfarin dose adjustment guideline
Ghana Standard Treatment Guidelines 2010^97^	“Warfarin, oral, adults: 10 mg daily at 6 pm for 2 days, then 5 mg daily”	“Regular dose adjustment and monitoring of INR until target of 2.0 and 3.0 is attained”
Namibia Standard Treatment Guidelines 2011^98^	“Warfarin: 5 to 10 mg orally; start at same time as heparin”	“Adjust dose according to INR”
Ethiopia Standard Treatment Guidelines for General Hospitals 2014^99^	“Warfarin (starting simultaneously with heparin), 5 mg orally, daily”	“Dose adjusted to achieve target INR of 2.0 to 3.0″
Uganda Clinical Guidelines. National Guidelines for Management of Common Conditions 2016^100^	“… plus warfarin 5 mg single dose given in the evening, commencing on the same day as heparin”	“Maintenance dose 2.5 to 7.5 mg single dose daily, adjusted according to the INR 2 to 3″
Kenya National Guidelines for Cardiovascular Diseases Management 2018^101^	“Recommended starting dose: 5 mg orally once a day”	“Typical maintenance dose: 2 to 10 mg orally once a day. Dosage must be individualized according to the patient's INR”
Standard Treatment Guidelines and Essential Medicines List for South Africa, Hospital Level, Adults 2019^102^	Day 1: 5 mg daily (2.5 mg daily for high sensitivity) 2‐3 d after initiation: • 5‐7.5 mg/d if INR < 1.5 • 2.5‐5 mg/d if INR 1.5 to 1.9 • 2.5 mg/d if INR 2.0 to 2.5 • hold warfarin if INR > 2.5 2‐3 d after last INR check: • 7.5‐10 mg/d if INR < 1.5 • 5‐10 mg/d if INR 1.5 to 1.9 • 2.5‐5 mg/d if INR 2.0 to 3.0 • hold warfarin if INR > 3	• Increase weekly dose by 10% if INR < 1.5 • Increase weekly dose by 5% if INR 1.5 to 1.9 • No change to warfarin dose if INR 2.0 to 3.0 • Decrease weekly dose by 5% if INR 3.1 to 4.0 • Decrease weekly dose by 10% if INR 4.1 to 5.0 • Decrease weekly dose by 20% if INR 5.1 to 9.0

INR, international normalised ratio.

Where detailed dose adjustment algorithms do not exist, clinicians may make erroneous or even paradoxical dose adjustments. Reviews from Cameroon,^8^ Ethiopia^9^, ^14^ and Namibia^19^ report dose increases after 4‐17% of supratherapeutic INRs and dose decreases after 4‐15% of subtherapeutic INRs. In addition, dose adjustments are often inappropriately large: While clinical trial evidence has shown that warfarin dose adjustments of 10‐15% were associated with better outcomes,^103^ the mean warfarin dose increase in one Ethiopian review was 58% in response to an INR of 1.5‐1.9 against a target of 2.0‐3.0.^9^ In the Namibian example, more than half of patients with an INR >4 were over‐corrected so that their subsequent INR was subtherapeutic.^19^

Warfarin formulations other than 5 mg are frequently not available in SSA,^6^, ^22^ making precision dosing difficult. Complicated weekly dosing schedules of alternating daily dosages which often require tablet splitting (sometimes into quarters) are used, potentially compromising the actual dose taken.^104^ This may in turn influence adherence and anticoagulation control. Reasons for the unavailability of alternative formulations seem to be market‐related as several SSA countries' essential medicines lists follow the WHO model list, which includes 1, 2 and 5 mg warfarin tablets. Even so, some Kenyan evidence suggests prescribers may simply be unaware of the market availability of alternatives to 5 mg tablets.^83^ Also, 1 or 2 mg formulations may be priced close to 5 mg tablets, making these alternative formulations less cost‐effective.

## 6 PATIENT‐RELATED CHALLENGES

The importance of pharmacogenetic variability on warfarin dose requirements in SSA was demonstrated in a recent systematic review.^105^ For example, three variants that are more prevalent in Black Africans than in other populations, CYP2C9*5, CYP2C9*6 and CYP2C9*11, affected warfarin dose requirements by −13, −8 and −5 mg/week, respectively.^105^

Potential drug‐drug interactions between warfarin and co‐prescribed medications hinder good anticoagulation control. In SSA, antiretroviral therapy is a significant source of potential drug‐drug interactions.^18^, ^20^, ^22^, ^70^, ^92^, ^93^, ^106^ Tuberculosis is also common in SSA, and rifampicin use induces multiple cytochrome P450 isoforms, resulting in a reduced warfarin effect. In a Kenyan case series of patients on concurrent rifampicin and warfarin, the median warfarin dose increase with rifampicin was 16%, but some patients required dose increases up to +441%.^107^ In a Ugandan case series, patients concomitantly prescribed rifampicin, antiretroviral therapy and warfarin had highly labile INRs and warfarin dose requirements, and it was not possible to predict the course of INR results in any individual patient.^108^ In Kenya, VTE patients with advanced HIV and tuberculosis required a median eight additional clinic visits to achieve or maintain a therapeutic INR.^109^

Despite widespread herbal medicine use in SSA^110^, ^111^ there is very little data on how this influences anticoagulation.^112^, ^113^ Nevertheless, it is plausible that some herbal medicines may interact with warfarin.

Anticoagulation patients in SSA are younger than those in high‐income settings. For example, in Uganda and South Africa the median age of patients attending five anticoagulation services was 56 years^22^ and in a Kenyan service the mean age was 43 years.^20^ Younger patients often show reduced adherence compared to older patients.^114^ Two possible reasons for this are that they are economically active and therefore may be unable to attend follow‐up appointments, and that they may have reproductive wishes and expectations and therefore intentionally reduce their intake of a potentially teratogenic medicine.^115^, ^116^

Four studies reporting patients' self‐reported adherence to anticoagulants in SSA are summarised in Table 4. Notably, from these, fewer than half of patients considered themselves highly adherent to warfarin. One study suggested that warfarin nonavailability may contribute to poor adherence.^64^ In an analysis of the “care cascade” of rheumatic heart disease patients in Uganda, retention in care was the stage with the highest patient drop‐out.^48^

**TABLE 4:** Sub‐Saharan African studies of patients' self‐reported adherence to anticoagulation

Study	Setting, sample	Adherence measure	Outcome
Chalachew^29^	Addis Ababa, Ethiopia, 2019 Children and young adults (11‐25 yr) with prosthetic valves on anticoagulation at a teaching hospital	Not reported	30/73 (41%) reported perfect adherence, 34/73 (47%) missed 1‐2 doses per week. 9/73 (12%) missed >2 doses per week Most common reasons were forgetfulness (30%) and unavailability of warfarin (23%) Association between adherence and INR control not reported
Mariita^115^	Nairobi, Kenya, 2015 Consecutive sample at cardiac, cardiothoracic and haemato‐oncology clinics of a teaching hospital	MMAS‐8^117^ High adherence if score = 8, moderate adherence if score 6 or 7, low adherence if score <5	77/147 (52%) high adherence, 53/147 (36%) moderate adherence, 17/147 (12%) low adherence Association between adherence and INR control not reported
Iqbal^118^	Nairobi, Kenya, 2017 Convenience sample at cardiac, cardiothoracic haemato‐oncology and DVT clinics of a teaching hospital	MMAS‐8^117^ High adherence if score = 8, moderate adherence if score 6 or 7, low adherence if score <5	13/45 (29%) high adherence, 32/45 (71%) low adherence Association between adherence and INR control not reported
Eltayeb^119^	Khartoum, Sudan, 2017 Convenience sample at cardiothoracic clinic of a teaching hospital	Four‐item MMAS^120^ Considered adherent if score = 0, nonadherent if score >0	5/93 (5.4%) adherent Association between adherence and INR control not reported

INR, international normalised ratio; MMAS‐8, eight‐item Morisky medication adherence scale.

We are not aware of any studies reporting SSA patients' attitudes and beliefs about anticoagulation. A few studies (Table 5) reported on patients' anticoagulation knowledge, with generally low levels of knowledge found. Only two studies investigated whether participants' anticoagulation knowledge correlated with their anticoagulation control and these reached conflicting results.^10^, ^121^ Levels of knowledge were generally associated with participants' level of education and with the provision of written educational materials. Topics on which participants' knowledge was low were drug and food interactions, the effect of missing a dose, the interpretation of INR values, recognizing the symptoms of over‐ or underdosing, contraception and pregnancy planning.^10^, ^82^, ^122^

**TABLE 5:** Sub‐Saharan African studies of patients' knowledge about anticoagulation

Study	Setting, sample	Knowledge measure	Outcome
Dwamena^121^	Accra, Ghana, 2012 Systematic sample of outpatients at anticoagulation clinic of a teaching hospital	Own tool, adapted from Taylor,^123^ pass rate set at 70%	112/175 (64%) passed Better knowledge was associated with better INR control
Assefa^82^	Addis Ababa, Ethiopia, 2014 Outpatients on warfarin at a teaching hospital	Own tool, adapted from AKA,^124^ pass rate set at 75%	18/130 (14%) passed Mean score was 59% Association between knowledge and INR control not reported
Mariita^10^	Nairobi, Kenya, 2016 Consecutive sample at cardiac, cardiothoracic and haemato‐oncology clinics of a teaching hospital	Own tool, adapted from OAK,^125^ pass rate set at 75%	15/147 (10%) passed Mean score was 57% Knowledge was not associated with INR control
Iqbal^118^	Nairobi, Kenya, 2017 Convenience sample at cardiac, cardiothoracic, haemato‐oncology and DVT clinics of a teaching hospital	Own tool, adapted from OAK,^125^ “satisfactory knowledge” set at >70%	12/45 (27%) had satisfactory knowledge pre‐intervention Association between knowledge and INR control not reported
Hutheram^126^	Gauteng, South Africa, 2016 Convenience sample at 10 private sector INR clinics attached to a private pathology company	Own tool, adapted from OAK,^125^ pass rate set at 50%	31/34 (91%) passed Association between knowledge and INR control not reported
Samadoulougou^127^	Ouagadougou, Burkina Faso, 2014 Convenience sample of patients in the cardiology clinic of a university hospital	Own tool, adapted from Janoly‐Duménil^128^	Participants scored low in questions relating to their ability to anticipate and make decisions in risky situations Association between knowledge and INR control not reported
Maramba^32^	Harare, Zimbabwe, 2018 Convenience sample of outpatients with thrombophilia on long‐term warfarin	Not clear	29/47 (62%) were not aware of the need for regular check‐ups Association between knowledge and INR control not reported
Gregersen^122^	Johannesburg, South Africa, 2006 Convenience sample of women of childbearing age with valvular heart disease who had at least one pregnancy while on warfarin	Own questionnaire	38/124 (31%) were not using contraception; misperceptions about “the contraceptive effect of warfarin” were not uncommon Knowledge about effects of warfarin on the foetus was often inaccurate and not specific or detailed Association between knowledge and INR control not reported

AKA, anticoagulation knowledge assessment; INR, international normalised ratio; OAK, oral anticoagulation knowledge test.

## 7 OVERCOMING THE CHALLENGES

Overcoming the multitude of challenges faced by anticoagulation services and patients requiring anticoagulation in SSA require a multifaceted approach. “Warfarin care bundles” are effective and viable strategies, as shown in a recent network meta‐analysis of anticoagulation interventions.^129^ In SSA such a warfarin care bundle must include both process‐centred and patient‐centred activities, the exact combination of which will be specific to each setting and will depend on cost‐effectiveness to guide rational allocation of limited resources. In making process changes, it will be important to leverage off existing successful systems, such as HIV treatment programmes,^130^ and to ensure these changes are embedded in a quality improvement framework, with regular feedback to patients, clinicians and managers.

Providing patient‐centred anticoagulation education and adherence support are two interventions possibly achievable over a shorter term. These interventions have shown benefit on patients' knowledge, adherence and INR control in a few individual SSA studies^13^, ^14^, ^118^ and may be of particular benefit in vulnerable populations.^131^ As pharmacists and doctors are a scarce resource in Africa, these education and support tasks can be successfully shifted to mid‐level healthcare workers.^7^ SSA has extensive expertise in providing adherence support to patients with HIV, and education and adherence support for patients with noncommunicable diseases should build on these existing systems.^132^

Process‐centred activities are likely to require a longer term, and more resources, to implement successfully. These activities may include decentralisation of anticoagulation services, setting up of anticoagulation clinics, improving access to warfarin (including formulations other than the 5 mg tablet), improving access to laboratory testing and/or scaling up point‐of‐care INR testing, task‐shifting of anticoagulation care to mid‐level healthcare workers, staff training, and implementing locally validated dose initiation and dose adjustment algorithms. Decentralised anticoagulation clinics can be successfully implemented in SSA, with improved outcomes (and better cost‐effectiveness) compared to those achieved at a central referral hospital.^93^ Point‐of‐care INR testing has also been successfully implemented in SSA, drastically reducing the number of visits patients have to make to the clinic.^87^ However, the high cost of test strips is problematic, and relying on donations and subsidies is not sustainable. Localised dose initiation and dose adjustment algorithms must consider the comorbidities and potential drug interactions that are prevalent in SSA, such as HIV, tuberculosis, antiretrovirals, antituberculosis therapy, co‐trimoxazole and herbal/traditional medicines. These algorithms must be easy to implement, for example be paper‐based, and should recommend small, percentage‐based dose adjustments.^103^

One example of an effective anticoagulation programme combining multiple interventions has come from Rwanda.^133^ In this programme, specialist noncommunicable disease nurses deliver postoperative care to valvular heart disease patients in decentralised clinics. Standard dosing algorithms are used, while nurses are supervised and supported by cardiologists, using mobile communications. Adherence support, as well as financial support, is offered to patients. While this small study did not report the effect of this programme on TTR, low mortality was described and there were no bleeding or thrombotic complications.^133^

DOACs may be a solution to some anticoagulation challenges in SSA: while they are still prohibitively expensive to most African patients and healthcare systems, they will in the future come off patent and generics may be more affordable. DOACs have the benefit of being used at fixed rather than individualised doses for the majority of patients and do not require routine monitoring.^73^ However, DOACs come with their own set of challenges. Fixed DOAC doses may not be appropriate for all patients, and some patients should be monitored for safety and efficacy.^134^, ^135^, ^136^ In addition, DOACs are contraindicated in valvular heart disease, a significant group of patients in SSA, and require dose adjustment/are contraindicated in severe renal impairment. DOACs have shorter half‐lives than warfarin, making strict adherence more critical; ironically, without regular monitoring, adherence problems may be missed in patients on DOACs.^73^ DOACs are also subject to drug‐drug interactions: notably for SSA, these include interactions with rifampicin and many antiretroviral agents.^137^ Finally, the management of major bleeding occurring with DOACs will require specific protocols, localised for SSA.^73^

## 8 CONCLUSION

There is significant room for improvement of anticoagulation control in countries across SSA. Increasing numbers of patients will need anticoagulation in the future because of the increasing burden of noncommunicable disease in the region. Despite the many challenges faced by patients, healthcare providers and health systems in these resource‐limited settings, several opportunities for improvement exist. Decentralisation of anticoagulation care, together with expanded access to medicines and monitoring, and enhanced support to practitioners and patients, are pivotal in achieving better control. Dose initiation and dose adjustment protocols that have been developed taking locally relevant factors into account can also contribute to better anticoagulation control. With the cost of DOACs still prohibitive, locally developed “warfarin care bundles” which address multiple anticoagulation challenges in combination, particularly when they leverage off systems that are already functional in SSA, currently appear to be the most appropriate strategy to improve anticoagulation control in this setting.

### 8.1 Nomenclature of targets and ligands

Key protein targets and ligands in this article are hyperlinked to corresponding entries in **http://www.guidetopharmacology.org**, the common portal for data from the IUPHAR/BPS Guide to PHARMACOLOGY, and are permanently archived in the Concise Guide to PHARMACOLOGY 2019/20.^138^

## COMPETING INTERESTS

M.P. receives research funding from various organisations including governmental bodies such as the MRC, the EU Commission and Health Education England. M.P. also received partnership funding for the following: MRC Clinical Pharmacology Training Scheme (co‐funded by MRC and Roche, UCB, Eli Lilly and Novartis), a PhD studentship jointly funded by EPSRC and Astra Zeneca, and grant funding from Vistagen Therapeutics. M.P. also received unrestricted educational grant support for the UK Pharmacogenetics and Stratified Medicine Network from Bristol‐Myers Squibb and UCB. M.P. held a joint grant from NIHR with MC Diagnostics for the development of an HLA panel. None of the funding M.P. received is related to the current paper.

The remaining authors declare no conflicts of interest.

## CONTRIBUTORS

This manuscript was conceptualised by all the authors. J.P.M. conducted the search, analysed the data, interpreted the data and wrote the first draft of the manuscript. All authors critically revised the manuscript for important intellectual content.
